# Retraction Note: Acetyl-CoA synthetase 3 promotes bladder cancer cell growth under metabolic stress

**DOI:** 10.1038/s41389-023-00484-0

**Published:** 2023-07-26

**Authors:** Jianhao Zhang, Hongjian Duan, Zhipeng Feng, Xinwei Han, Chaohui Gu

**Affiliations:** 1 Department of Interventional Radiology, First Affiliated Hospital of Zhengzhou University, Zhengzhou City, Henan Province 450052 China; 2 Department of Urology, Henan Institute of Urology and Zhengzhou Key Laboratory for Molecular Biology of Urological Tumor Research, First Affiliated Hospital of Zhengzhou University, Zhengzhou City, Henan Province 450052 China

**Keywords:** Cancer, Cell biology

Retraction to: *Oncogenesis* 10.1038/s41389-020-0230-3, published online 12 May 2020

The Editor-in-Chief has retracted this article. Concerns were raising regarding Figure 2H. This figure appears to overlap with Figure 3G in an article by different authors that was simultaneously under consideration in another journal [1].

The authors have provided partial raw data to address this concern. However, they have stated that some of the original data are no longer available. The Editor-in-Chief therefore no longer has confidence in the presented data.

All authors agree to this retraction.
